# Association Between Heart Rate Variability and Decompression-Induced Physiological Stress

**DOI:** 10.3389/fphys.2020.00743

**Published:** 2020-07-03

**Authors:** Sergio Rhein Schirato, Ingrid El-Dash, Vivian El-Dash, Bruna Bizzarro, Alessandro Marroni, Massimo Pieri, Danilo Cialoni, José Guilherme Chaui-Berlinck

**Affiliations:** ^1^Department of Physiology, Biosciences Institute, University of São Paulo, São Paulo, Brazil; ^2^Peter Murányi Experimental Research Center, Albert Einstein Hospital, São Paulo, Brazil; ^3^DAN Europe Research Division, Roseto degli Abruzzi, Italy; ^4^Environmental Physiology and Medicine Laboratory, Department of Biomedical Sciences, University of Padova, Padua, Italy

**Keywords:** heart rate variability, hyperbaric environments, decompression, decompression profiles, decompression sickness, endothelial function, inflammation, immune system

## Abstract

The purpose of this study was to analyze the correlation between decompression-related physiological stress markers, given by inflammatory processes and immune system activation and changes in Heart Rate Variability, evaluating whether Heart Rate Variability can be used to estimate the physiological stress caused by the exposure to hyperbaric environments and subsequent decompression. A total of 28 volunteers participated in the experimental protocol. Electrocardiograms were performed; blood samples were obtained for the quantification of red cells, hemoglobin, hematocrit, neutrophils, lymphocytes, platelets, aspartate transaminase (AST), alanine aminotransferase (ALT), and for immunophenotyping and microparticles (MP) research through Flow Cytometry, before and after each experimental protocol from each volunteer. Also, myeloperoxidase (MPO) expression and microparticles (MPs) deriving from platelets, neutrophils and endothelial cells were quantified. Negative associations between the standard deviation of normal-to-normal intervals (SDNN) in the time domain, the High Frequency in the frequency domain and the total number of circulating microparticles was observed (*p*-value = 0.03 and *p*-value = 0.02, respectively). The pre and post exposure ratio of variation in the number of circulating microparticles was negatively correlated with SDNN (*p*-value = 0.01). Additionally, a model based on the utilization of Radial Basis Function Neural Networks (RBF-NN) was created and was able to predict the SDNN ratio of variation based on the variation of specific inflammatory markers (RMSE = 0.06).

## Introduction

Several reports over the past two decades have shown that exposure to hyperbaric environments and subsequent decompression have many physiological implications, including reduction in endothelial function, activation of the immune system (Brubakk et al., 2005; Thom et al., 2012, 2015) and the well documented bubbles formation. The causal relationship between venous gas emboli formation and other decompression-related physiological alterations, is yet to be understood (Møllerløkken and Brubakk, 2009) and due to large inter-personal variability (Papadopoulou et al., 2018), venous gas emboli are a poor surrogate for decompression sickness (Doolette, 2016) on an individual basis.

In recent years the endothelial dysfunction hypothesis (Madden and Laden, 2009), which theorizes that microparticles associated with endothelial damage may act as nucleation sites for bubble formation, has drawn attention and gained support. This hypothesis is based on the recently accumulated knowledge about small particles (microparticles or microvesicles) shed by different cells in an organized and regulated manner. Such microparticles (MPs), which carry various nuclear components of their originating cells, like RNA and DNA, are involved in cell signaling and communication, and have recently emerged as relevant markers of inflammatory diseases (Batool et al., 2013). Variations in their levels and the cells from where they originated have been linked to an increasing range of diseases and inflammatory processes (Cognasse et al., 2015) (Puddu et al., 2010). This has led to decompression sickness being seen not as merely a physical or mechanical problem, but instead as a result of a complex biochemical process.

One study has shown that the exposure to high-pressure environments, even in the absence of decompression, is sufficient to increase the production of MPs carrying IL-1β (Stephen et al., 2018) an interleukin that belongs to cytokines family, which is an important mediator in inflammatory responses. The same study demonstrated that individuals exposed to higher ambient pressures produced a higher average count of MPs after the dive, which persisted for at least 2 h after the end of the dive, while the changes observed in the group exposed to lower ambient pressure resolved within 2 h after the dive.

The mechanism behind the formation of such microparticles has been described as related to high inert gas pressure, which causes singlet oxygen formation, a potentially toxic free radical initiated by a cycle of actin S-nitrosylation, nitric oxide synthase-2, and NADPH oxidase activation (Thom et al., 2014).

The NADPH oxidase, due to neutrophils activation, causes the generation of reactive oxygen species (ROS) that are originated by their heme enzyme myeloperoxidase (MPO) (Winterbourn et al., 2016). Exposure to high inert gas pressures, even in the absence of decompression, is apparently linked to an increased production of ROS. Despite their harmful effects to the host, the production of ROS is part of an orchestrated physiological response of the immune system to halt bacteria and fungus growth. Therefore, it is expected that higher expressions of MPO are linked to the generation of ROS and ultimately, due to the mechanism described above, microparticles. In fact, it has been demonstrated that individuals suffering from decompression sickness present higher levels of circulating MPs, as well as higher levels of MPO expression, for long periods after the appearance of the symptoms, although altered levels of such markers are also observed in symptom free individuals that were submitted to hyperbaric exposure. It seems, however, that in asymptomatic individuals the time for the resolution of the alterations is shorter and the change magnitude is lower (Thom et al., 2015).

Besides the well documented appearance of bubbles, alterations in the endothelial function and biochemical markers found in the blood stream, recently published studies also reported changes in the Heart Rate Variability (HRV) after exposure to hyperbaric environments (Noh et al., 2018; Schirato et al., 2018).

Heart Rate Variability is defined as the undirected changes in the interval between successive normal heartbeats. Usually, this is assessed through the timing between QRS complexes in a continuous electrocardiograph recording (ECG). It is the result of the balance between the sympathetic and the parasympathetic branches of the autonomic nervous system (ANS) (American, 1996), as well as of other non-neural sources of variation.

Heart Rate Variability is commonly studied in the time and frequency domains, and eventually through the application of non-linear methods, which will not be discussed in this study. Different HRV indicators have been associated with sympathetic or parasympathetic activity. High Frequency is highly impacted by the respiratory pattern, while Low Frequency is affected by both, the sympathetic and the parasympathetic branches of the ANS (Acharya et al., 2006). Additionally, an association between the Low Frequency and the baroreflex function has been made by at least one study (Rahman et al., 2011).

A reduction in HRV has been reported in several cardiological and non-cardiological diseases, ranging from diabetes to renal failure, to mention a few (Appel et al., 1989; Malliani et al., 1991; Sloan, 2015). A reduction in HRV, when analyzed in the frequency domain has also been associated with inflammatory processes (von Känel et al., 2008; Sloan, 2015).

The present study hypothesizes that changes in HRV might be related to decompression-induced physiological stress and, ultimately, with the endothelial and immune function alterations. However, HRV is highly unsteady and there is no *a priori* reason to expect that a given person would present similar values in different days, and so, there is an important variance in its estimators, such as the standard deviation of the interval between consecutive heartbeats (SDNN), both among and within subjects. As expected, a large inter-individual variance in the responses to decompression was observed in the present study. This, in addition to the fact that the study was produced with a relatively small cohort, leads to caution when the statistical significance of the results is analyzed. When observing the associations provided by the univariate regressions, or the correlations matrix, statistical significance values higher than 0.05 should not necessarily confirm or deny a specific relationship, but should be taken into context. In order to address this issue, Neural Networks structured as the sum of Radial Basis functions, a more robust technique for the construction of a predicting model, was adopted in the present study, with the final objective of evaluating the existence of a relationship between HRV and specific markers of decompression-induced physiological stress.

## Materials and Methods

The present study was undertaken in healthy individuals, all trained divers, experienced in the experimental profiles utilized. All volunteers provided a written informed consent. The ethical committee of the Biosciences Institute of the University of São Paulo approved the experimental protocol (CAAE #91231618.6.0000.5464).

### Simulated Dives

Experiments involving exposure to hyperbaric environment and subsequent decompression were conducted at three different facilities: (i) the hyperbaric chamber at the Centro Hiperbárico Paulista, (ii) the Brazilian Navy hyperbaric chamber at the Centro de Instrução e Adestramento Almirante Átilla Monteiro Aché (CIAMA) facility, and (iii) the Y-40 swimming pool located in Montegrotto Terme, Italy, under the coordination of the DAN Europe Foundation’s Research Division. All experiments were executed under the supervision of a trained physician.

Each volunteer underwent two different trials, both with the same maximum depth and bottom time, but with different decompression profiles. Decompression schedules were created to simulate one profile shallower stops and another with deeper stops while, keeping similar total decompression times. The results of this study will, however, be shown consolidated, disregarding the decompression profile used. Total inter gas supersaturation was computed and defined to be approximately equal in equivalent decompression profiles (i.e., shallower or deeper decompression stops), independently of the different participating facilities.

**FIGURE 1:**
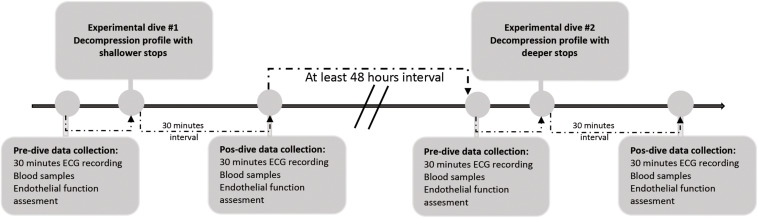
Experimental design flow chart.

Each trial was performed in the morning, at the same time of the day, and the interval between the experiments was at least 48 h for each volunteer, in order to minimize any carry-over effect (Cialoni et al., 2017). As for the time of the day, the only exceptions were the two experiments at the Y – 40 Swimming Pool that happened at different times, due to logistical challenges. A flow chart displaying the experimental design is illustrated in Figure 1.

The experiments executed at the Centro Hiperbárico Paulista were performed using electronically controlled closed-circuit rebreathers, while the experiments performed at the navy facility used breathing gas supplied through the chamber’s built-in breathing system (BIBS) and the volunteers that participated in the Y- 40 experiment used self-contained breathing apparatus (SCUBA).

### Electrocardiographic Data

Electrocardiograph records were obtained with superficial electrodes in a modified CM5 thoracic positioning. Data were collected while the subjects were seated in a comfortable position though MP36 systems (Biopac Systems, Inc.), set up at AHA configuration, with 0.05 Hz and 100Hz as low and high pass filters, respectively, and a sampling rate of 1000Hz.

There were two phases of continuous data collection: a 30-min pre-dive period used to establish the baseline condition for each volunteer and a 30-minutes post dive reading that was initiated 30 min after the end of the dive. This protocol was adopted due to previous observations that the magnitude of the changes in HRV tends to be higher in the second half-hour post decompression (Schirato et al., 2018). Interestingly, it is well documented that venous gas bubbles counts tend to take approximately the same amount of time to reach a peak (Cialoni et al., 2017).

Electrocardiograph recordings were converted into R-R intervals. Each of these time-series was, then, subdivided into non-overlapping windows of 256 consecutive R-R intervals. Subsequently, the following estimators of HRV were obtained from each R-R window (as detailed in American, 1996):

Time domain:

– R–R interval.
– SDNN. Standard deviation of the normal-to-normal R-R interval.
– RMSSD. The square root of the mean squared differences of successive R-R intervals.

Frequency domain:

– Fast Fourier Transform (FFT), to obtain the Power Spectrum Density (PSD) (Task Force, 1996), which was subsequently divided in:
  1. Ultra-low Frequencies: 0.01 to 0.04 Hz, not relevant to this study, due to the relatively short ECG recording intervals.
  2. LF: 0.04 to 0.15 Hz.
  3. HF: 0.15 to 0.4 Hz.

As the size of the time-series tends to infinity, the variance and the total power of the spectrum converge to each other, for this reason, in the present study, the power of a given PSD was approximated by SDNN^2^.

LF and HF power were also computed as fractions of the total power, as defined above (i.e., SDNN^2^) and in normalized units (nu), where the respective frequency was divided by the sum of all calculated frequencies up to 0.4 Hz. As an example, LF n.u. is equal to LF divided by the sum of VLF, LF and HF, multiplied by 100.

All data computational treatment was made through a set of implemented scripts in MatLab suite (MathWorks Inc.) and in R language.

### Blood Samples

Venous blood was collected from an antecubital arm vein by a trained phlebotomist before and after each (simulated) dive. The following variables were measured: red blood cells, hemoglobin, hematocrit, neutrophils, lymphocytes, platelets, aspartate transaminase (AST), alanine transaminase (ALT), immunophenotyping, and microparticles for quantification through Flow Cytometry (see below). These last samples were collected using tubes Cyto-Chex BCT (Streck, INC.).

Blood samples were drawn immediately before the experiment and one hour after the end of decompression. Blood works and liver function analyses were performed immediately after collection at the hyperbaric center. Immunophenotyping was performed up to 10 days after the blood collection (in the case of the experiments in Italy), although the average interval between collection and analysis was 2 days (in all other cases).

### Flow Cytometry

Immunophenotyping studies were performed with a 16-color FACSFortessa^TM^ (Becton & Dickinson Company©, BD) using manufacturers’ acquisition software.

Annexin binding buffer and the following antibodies were purchased from Biolegend (San Diego, CA, United States): fluorescein isothiocyanate FITC-conjugated anti-annexin V, FITC-conjugated anti-human myeloperoxidase (MPO), APC-conjugated anti-human CD41, PerCF594-conjugated anti-human CD14, PerCP-conjugated anti-human CD235, Pacific Blue-conjugated anti-human CD31, AF700-conjugated anti-human CD66b, and APC- conjugated anti-human CD19. Additionally, Live/Dead V-500 conjugated anti-human was used to define the dead cells population. Immunophenotyping through flow cytometry to evaluate the populations of granulocytes (CD 16 + /CD66b +) among live cells was performed, while controlling for the percentage of granulocytes expressing myeloperoxidase on its surface (MPO%) and mean fluorescence intensity of myeloperoxidase (MPO MFI), as indicators of neutrophil activation. The strategy used in this analysis and the hierarchy of the gates is detailed in Appendix I.

For MPs acquisition and processing, blood was centrifuged for 5 min at 1,500 *g* (Thom et al., 2013b). The supernatant was centrifuged at 15,000 *g* for 30 min to pellet the few remaining platelets and cell debris. These samples were then frozen at minus 80°C, allowing them to be analyzed on a future date. Studies were performed with a 16-color Moflo Astrios (Beckman Coulter, Brea, CA, United States) using manufacturers’ acquisition software. Annexin binding buffer was used in the absence of any other marker, so that forward and side scattering (FSC and SCC, respectively) voltages could be increased enough to visualize the electronic noise produced by the equipment, setting it to its highest sensitivity. A gate was designed around the noise to separate it from MPs. Polystyrene beads (Bang Beads, Beckman Coulter) with known sizes of 0.3, 1 and 3 μm were used for the voltage adjustment for MPs sizes and to set up the gates, delimiting MPs while excluding electronic noise. Annexin binding buffer in conjugation with each of the antibodies specified below, without the addition of samples containing microparticles, were tested separately to control for background fluorescence and unspecific signals. Antibody-stained MPs were used to assess each signal separately and confirm inexistence of overlap or interference between the channels. Samples were incubated for 30 min with 2 μL of each antibody, protected from light and then diluted to 2mL with annexin V binding buffer. Microparticles were assessed by Annexin V staining and discriminated by CD31, CD41 and CD66b markers to differentiate those derived from endothelial cells, platelets and granulocytes, respectively (Thom et al., 2012). Annexin binding buffer and the following antibodies were purchased from Biolegend (San Diego, CA, United States): fluorescein isothiocyanate FITC-conjugated anti-annexin V, Pacific Blue-conjugated anti-human CD31, APC-conjugated anti-human CD41 and AF700-conjugated anti-human CD66b. MPs were defined as Annexin V-positive particles with diameters up to 1.0 μm. and gates were set to include particles with sizes from 0.3 μm to 1.0 μm, with exclusion of background corresponding to debris usually present in buffers. Detergent Triton X was used as a control, as MPs are expected to disappear in its presence. Each sample analysis was performed using the software FlowJo Treestar © (FlowJo, Becton and Dickinson Company©, BD) at the Center for Experimental Research of the Hospital Albert Einstein.

### Volunteers

Twenty-eight civilian and military divers participated in this study (one volunteer participated in two experimental rounds) (Age = 43.6 ± 6.5 years, weight = 87 ± 12.4 kg, height 179 ± 7.9 cm, BMI 26.9 ± 3.5 Kg/m^2^). Three volunteers participated in only one experiment, being released from the second due to medical conditions not related to dive (in two cases) and in one case due to the development of decompression sickness in the first experiment. Blood samples for the quantification of red cells, hemoglobin, hematocrit, neutrophils, lymphocytes, platelets and for the assessment of liver function alterations (AST and ALT) were not obtained from all volunteers, therefore for each the result herein reported, the respective number of samples (*n*) will be reported. Volunteers data is given as the mean ± standard deviation.

### Statistical Analysis

Differences between pre and post dive data were determined using Student’s *t*-test for paired samples for the cases where a normal distribution of the observations was verified. When normal distribution was not confirmed, a non-parametric permutation test with 10.000 simulation rounds was performed for the definition of the *p*-value (Ludbrook and Dudley, 2008). The limit of significance was set at 0.05. Unless otherwise specified, all data provided in this study is given as the mean ± standard error (S.E.) The associations of HRV indicators SDNN, LF and HF with each of the markers analyzed were separately tested with linear regression models and the respective coefficients and *p*-values was displayed. A Pearson correlation coefficient matrix was calculated for all markers and HRV indicators in the time domain.

### Modeling

Modeling was done using a Radial Basis Function Neural Networks (RBF-NN), as detailed below, univariate and multivariate regressions. RBF-NN and multivariate were used in order to allow the comparison of the accuracy produced by each one and, since the RFB-NN provided a much higher accuracy, all the results obtained through multivariate regression were discarded and will not be discussed in this paper. The dataset was structured as a matrix *G*′ composed by elements $x_{k,h}^{\prime }$, where:

*h* represents the *h*^*th*^ variable (*h* = 1, 2,…, n),

k represents the *k*^*th*^ volunteer (*k* = 1, 2,…, N)

n is equal to the number of observed variables

N is equal to the number of volunteers.

Data was treated so that all variables were normalized according to equation 15 below and used to create the normalized matrix G:

(1) $$x_{k,h}=\frac{x_{k,h}^{\prime }-\min\left(x_{h}^{\prime }\right)}{\max\left(x_{h}^{\prime }\right)-\min\left(x_{h}^{\prime }\right)},\text{where}:$$

*x*_*k,h*_ = the normalized variable

$$G=\left[\begin{array}{cccc} x_{11}\; & x_{12}\; & \cdots \; & x_{1\,n}\\ x_{21}\; & x_{22}\; & \vdots \; & \vdots \\ x_{31}\; & x_{32}\; & x_{k,h}\; & \\ \vdots \; & \vdots \; & & \\ x_{N\,1}\; & x_{N\,2}\; & & x_{N\,n} \end{array}\right]$$

Radial Basis Function Neural Networks was structured as the function ψ, given by the sum of Gaussian radial basis functions:

(2) $$\psi =\sum_{j=1}^{S}a_{j}\,\gamma_{j}+b,\mathrm{where}:$$

*a*_*j*_ = calculated coefficient for number of clusters j

b = adjustmemt coefficient

Where γ_*j*_ is given by:

(3) $$\gamma_{j}\,\left(x\right)=e^{-1/2\sigma^{2}∥x_{N\,1},x_{N\,2},\ldots ,x_{N\,n}-M_{j}{∥}^{2}},w\,h\,e\,r\,e:$$

j = number of clusters, where *j* = 1,2,…,*S*

*M*_*j*_ = centroids for any given cluster. *M*_*j*_ = [*m*_1,*j*_,*m*_2,*j*_,*m*_3,*j*_,…,*m*_*n*,*j*_], thus: *M*_*j*_ = {*m*_*h*,*j*_}

The centroids vector *M*_*j*_ were stochastically determined between [0, 1]. The Euclidean distance *E*_*k,j*_, given by the sum of the norms $∥x_{k,h}-M_{j}^{2}∥$, is equal to:

(4) $$E_{k,j}=\sum_{h=1}^{n}{\left(x_{k,h}-m_{h,j}\right)}^{2}$$

For the model used in this study one single sigma was calculated for all centroids, as per equation 5 below. The utilization of one sigma for each centroid might improve the accuracy of the model in certain cases (Haykin, 1999), but did not add additional accuracy in this case.

(5) $$\sigma =\frac{D_{\max}}{\sqrt[{2}]{2\,S}},\mathrm{where}:$$

*Dmax* = *maximum distance between any pair of centroids*

*S* = *number of centroids*

The calculated coefficients *a*_1_,*a*_2_,…,*a*_*j*_ and the adjustment coefficient *b*, used to construct the matrix *w*, were determined by solving the system below:

$$\underset{\theta }{\underbrace{\left[\begin{array}{cccc} e^{-\frac{E_{k,j}}{2\,\sigma^{2}}}\; & \cdots \; & e^{-\frac{E_{k,j}}{2\,\sigma^{2}}}\; & 1\\ \vdots \; & \ddots \; & \vdots \; & \vdots \\ e^{-\frac{E_{k,j}}{2\,\sigma^{2}}}\; & \cdots \; & e^{-\frac{E_{k,j}}{2\,\sigma^{2}}}\; & 1 \end{array}\right]}}\cdot \underset{W}{\underbrace{\left[\begin{array}{c} a_{1}\\ a_{2}\\ \vdots \\ a_{j}\\ b \end{array}\right]}}=\underset{y}{\underbrace{\left[\begin{array}{c} y_{1}\\ y_{2}\\ \vdots \\ y_{N} \end{array}\right]}}$$

Since θ is a not a square matrix, the pseudo-inverse matrix θ^−1^ was calculated, as follows:

(6) $$\theta^{-1}={\left(\theta^{t}.\theta \right)}^{-1}.\theta^{t}$$

θ^*t*^ is the transpose of θ

Therefore:

(7) $$W=\theta^{-1}.y$$

A total of 10.000 iterations is made for the calculation of the coefficients *a*_*j*_ and *b*_*j*_ and the set {*a*_*j*_,*b*_*j*_} that provides the highest accuracy in the training set is chosen for the model. It is important to note that the number of iterations was arbitrarily defined.

The optimal number of clusters was determined using K-means algorithm, calculated for each set of training data, given by the minimization of the distance *J*, given by:

(8) $$J=\sum_{i=1}^{k}\sum_{j=1}^{S}{∥x_{k,h}-M_{j}∥}^{2}$$

In order to reduce the arbitrariness usually associated with the determination of the number of clusters that will be used in construction of the RBF-NN, a decay curve approximation of the sum of the within cluster sum of distances (WS) was created, according to the formula below:

(9) $$W\,S_{n}=W\,S_{1}*j^{-\varepsilon },\mathrm{where}:$$

*WS*_*n*_ = sum of within distance for the number of clusters

ε = calculated delay coefficient

ε is calculated according to the linearized form of equation 9.

(10) $$\log\left(W\,S_{j}\right)=\log W\,S_{1}-\varepsilon *\log\left(j\right)$$

For the purposes of the later discussion, the accuracy of the model was defined as the Root Mean Squared Error (RMSE) of the predicted versus the observed SDNN Ratio.

Standard deviation of normal-to-normal intervals Ratio is calculated as the SDNN measured after the experiment divided by the SDNN measured before the experiment.

Due to the relatively small sample, two different models were created. In the first model, the RBF-NN was trained with the data obtained in both profiles from all volunteers (*N* = 47), with the objective of finding out whether a relationship between the SDNN Ratio and the ratios of the inflammation-related variables (CD16(%), CD66b (MFI), MPO(%), MPO (MFI), Anexxin +, MP CD66b +, MP CD31 + and MP CD41 + calculated as the post experiment values divided by the pre experiment values). For clarification purposes, training the RBF-NN means creating a matrix *G*, with *N* equal to 47 (all the volunteers available), and *n* equal to 7 (the variables mentioned above).

A second model was created, where the calibration of the RBF-NN was done using only a portion of the data (*N* = 37), chosen in a stochastic process. As in the first model, Gaussian functions were chosen for activation and the model was based on the neural network that produced the best accuracy for the training set in 100 trials, in a stochastic process of centroids determination. The model created was then applied to the remaining 10 observations in an out-of-sample validation process.

## Results

Three volunteers participated in only one experiment, being released from the second due to medical conditions not related to dive (in two cases) and in one case due to the development of decompression sickness in the first experiment. The case where decompression sickness was diagnosed was promptly treated according to the parameters of the United States Navy Recompression Treatment Table 4 by the facility physician, with complete recovery immediately after treatment.

### Heart Rate Variability

Heart Rate Variability was evaluated for all volunteers in both time and frequency domains. Although data was obtained in two different experimental protocols, as described in the Materials and Methods section, the analysis of pre and post results was consolidated, since there was no statistically significant difference between them. An overall increase in variability was observed in all situations (Table 1).

**TABLE 1:** Time and frequency heart rate variability indicators.

	Pre dive mean	SE	Post dive mean	SE	*p*-value
Low frequency (ms2)	451.56	44.31	594.11	71.64	0.002
Total low frequencies (ms2)	608.72	55.42	809.37	85.42	0.001
High frequency (ms2)	69.24	8.32	106.53	18.94	0.001
LF/HF ratio	8.50	0.82	8.21	0.74	0.280
LF as ratio of total variability	0.23	0.02	0.23	0.02	0.461
Total low frequencies (n.u.)	0.90	0.01	0.89	0.01	0.203
HF as ratio of total variability	0.03	0.002	0.04	0.00	0.144
RMSSD	19.95	1.02	25.36	1.90	0.000
SDNN (ms)	43.35	1.57	48.40	2.60	0.002

Frequency domain indicators LF, Total Low Frequencies (VLF + LF) and HF increased. LF as proportion of HF and as proportion of total variability did not show significant changes, neither did HF as proportion of total variability. In the time domain, SDNN and RMSSD showed significant increases in the post dive values. It is interesting to note that both, HF and LF ratios as proportion of total variability (approximated by SDNN^2^) did not present changes, nor did Total Low Frequencies (VLF + LF) expressed in normalized units. These results are compatible with the overall increase in variability expressed by SDNN, while the average participation of each frequency band in the overall frequency domain remains the same.

### Blood Assay

Blood assays were performed for 25 volunteers. Leucocytes, red blood cells, hematocrit, hemoglobin and platelets counts were different between pre and post dive measurements. Statistically significant reductions in red blood cells, hematocrits, hemoglobin and platelets were observed, concomitantly with increases in leucocytes counts (Table 2).

**TABLE 2:** Red cells, hematocrit, leukocyte, platelet counts and liver function indicators.

	Pre dive mean	SE	Post dive mean	SE	*p*-value
Leucocytes × 10^3^	7.59	0.28	8.02	0.30	0.006
Platelets × 10^5^	2.58	0.10	2.53	0.09	0.026
Red cells (abs)	5.26	0.0001	5.20	0.09	0.032
Hemoglobin (abs)	15.50	0.0002	15.29	0.24	0.002
Hematocrit (abs)	45.01	0.0007	44.52	0.67	0.068
AST	22.12	2.21	22.96	2.34	0.154
ALT	38.35	6.16	38.73	6.17	0.597

### Liver Function

Enzymes aspartate transaminase (AST) and alanine transaminase, also referred to as alanine aminotransferase (ALT) were measured pre and post dive for 12 volunteers, in order to monitor for liver function alterations due to decompression. No significant increases were observed in either case, as detailed in Table 2.

### Flow Cytometry

Immunophenotyping through flow cytometry to evaluate the populations of granulocytes (CD 16 + /CD66b +) among live cells was performed, while accounting for the percentage of granulocytes expressing myeloperoxidase on its surface (MPO%) and the mean fluorescence intensity of myeloperoxidase (MPO MFI), as indicators of neutrophil activation (Table 3). An increase in the total population of granulocytes was observed (in line with the increased leucocytes observed in Table 2), while the MPO expression and the percentage of cells expressing MPO post dive values did not show significant changes in comparison with pre dive values.

**TABLE 3:** Granulocytes and MPO expression.

	Pre dive mean	SE	Post dive mean	SE	*p*-value
CD16 + /CD66 +	11.73	1.41	13.39	1.40	0.01
MPO + (%)	2.52	0.69	2.45	0.25	0.36
MPO (MFI)	459.80	96.95	421.33	78.36	0.16

**FIGURE 2:**
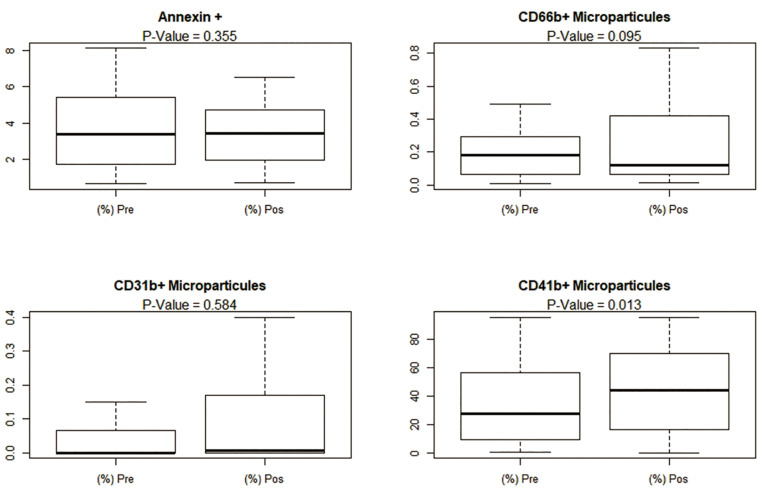
Pre dive and post dive numbers of circulating microparticles.

Microparticles were assessed by Annexin V staining and discriminated by CD31, CD41 and CD66b markers to differentiate those derived from endothelial cells, platelets and granulocytes, respectively. The microparticles population of was defined as positive for Annexin and analyzed as previously described, using microbeads with diameters of 0.3 μm and 1.0 μm to carefully assess the size of particles. Pre and post dive microparticles counts are displayed in Figure 2. An overall increase in the numbers of circulating microparticles was observed, but only microparticles positive for CD66b and CD41 presented significant changes.

### Linear Regressions and Correlations Matrix

Univariate regressions (*n* = 47) between SDNN, LF, HF, and immune system action markers obtained through flow cytometry were made (Table 4) and the coefficient (estimate) and *p*-values were calculated.

**TABLE 4:** Relationship between HRV indicators, MPO and microparticles.

	SDNN		LF		HF	
			
	Estimate	*p*-value	Estimate	*p*-value	Estimate	*p*-value
CD16 +	–22.89	0.09	–5.11	0.24	–0.94	0.25
MPO (%)	–2.68	0.91	1.08	0.89	–1.66	0.25
MPO (MFI)	0.44	0.01	0.07	0.23	0.01	0.28
Annexin +	–125.37	0.03	–25.45	0.16	–7.70	0.02
CD66b +	195.27	0.74	376.92	0.04	34.12	0.33
CD31 +	78.87	0.72	122.34	0.08	2.00	0.88
CD41 +	3.14	0.52	1.83	0.23	0.09	0.74

Standard deviation of normal-to-normal intervals showed significant negative correlation with the number of circulating Annexin + microparticles (*p*-value = 0.03) and a positive correlation with mean fluorescence intensity of myeloperoxidase (*p*-value = 0.01). A non-significant negative correlation between SDNN and the number of circulating granulocytes was also observed and will be discussed later. As it would be expected due to the high correlations between HF and SDNN, HF also demonstrated a negative correlation with the number of circulating Annexin + microparticles. LF has a positive association with CD66b + and CD31 + MPs, although not statically significant for the second, which will also be developed further in the section “DISCUSSION.”

A correlation coefficient matrix, correlating HRV indicators in the time domain and the variables obtained in the blood assay and the flow cytometry, and its respective *p-value*, was calculated (Table 5). These coefficients were calculated for the data set for which all variables of interest were collected (*n* = 37), therefore the results displayed in Table 5 are not necessarily comparable to the results displayed in Table 4.

**TABLE 5:** Pearson correlation analysis.

	**CD66b MFI**	**MP CD31 +**	**MP CD41 +**	**MP CD66b +**	**MPO (MFI)**	**Neutrophils**	**Platelets**	**RMSSD**	**SDNN**
Anexx +	0.473	−0.121	−0.2	−0.171	0.709	−0.457	−0.495	−0.291	−0.383
	*p*-value: 0.0031**	*p*-value: 0.474	*p*-value: 0.2353	*p*-value: 0.3129	*p*-value: 0***	*p*-value: 0.0045**	*p*-value: 0.0018**	*p*-value: 0.0803	*p*-value: 0.0193*
CD66b MFI		0.229	−0.373	−0.068	0.373	−0.663	−0.625	−0.057	−0.175
		*p*-value: 0.172	*p*-value: 0.023*	*p*-value: 0.6872	*p*-value: 0.023*	*p*-value: 0***	*p*-value: 0***	*p*-value: 0.7373	*p*-value: 0.2997
MP CD31 +			−0.111	0.129	−0.244	0.096	0.068	0.004	−0.083
			*p*-value: 0.5121	*p*-value: 0.4459	*p*-value: 0.1463	*p*-value: 0.5724	*p*-value: 0.6898	*p*-value: 0.9832	*p*-value: 0.6241
MP CD41 +				0.226	−0.409	0.542	0.585	0.152	0.22
				*p*-value: 0.1778	*p*-value: 0.0119*	*p*-value: 0.0005***	*p*-value: 0.0001***	*p*-value: 0.3678	*p*-value: 0.1917
MP CD66b +					−0.25	0.177	0.199	0.258	0.244
					*p*-value: 0.1363	*p*-value: 0.2946	*p*-value: 0.2368	*p*-value: 0.1225	*p*-value: 0.1448
MPO (MFI)						−0.55	−0.652	−0.284	−0.28
						*p*-value: 0.0004***	*p*-value: 0***	*p*-value: 0.0886	*p*-value: 0.0938
Neutrophils							0.4866	0.03	0.163
							*p*-value: 0***	*p*-value: 0.8593	*p*-value: 0.3352
Platelets								−0.052	0.001
								*p*-value: 0.7579	*p*-value: 0.9942
RMSSD									0.789
									*p*-value: 0***

Correlation coefficients and *p*-values are shown for the data set for which all information was available. *p-value < 0.5, **0.01 > *p*-value > 0.001, ***p-value < 0.001.

As it can be noted, SDNN is inversely correlated with the number of circulating Annexin + microparticles (as demonstrated in Table 4), and, as expected with RMSSD. A strong positive correlation is also present between the mean fluorescence intensity of myeloperoxidase and the number of circulating Annexin + microparticles. Additionally, the number of platelets negatively correlates with the mean fluorescence intensity of myeloperoxidase (which would be expected due to the platelet activation mechanism driven by MPO (Gorudko et al., 2013)), and with the mean fluorescence intensity of CD66b +.

### Modeling

**FIGURE 3:**
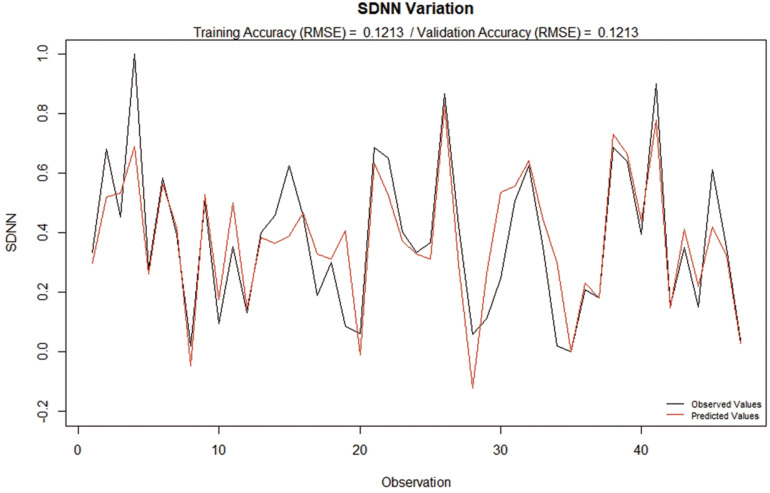
Observed and calculated SDNN Ratio based on the variation of CD16(%), CD66b (MFI), MPO(%), MPO (MFI), Annexin +, MP CD66b +, MP CD31 + and MP CD41 +. It is important to note that Training Accuracy and Validation Accuracy are the same, due to the fact that the model is being applied on the training set (in-sample validation). This test was done to evaluate how effective the algorithm is in reproducing the data used for calibration.

As described in section “MATERIALS AND METHODS,” a model was made using RBF-NN to estimate the variation of the SDNN Ratio (Figure 3) as a function of the variation of the CD16(%), CD66b (MFI), MPO(%), MPO (MFI), Annexin +, MP CD66b +, MP CD31 + and MP CD41 +, produced a Training Accuracy of 0.16 (expressed as RMSE), demonstrating that it is possible to use the variation of the observed variables to explain the variation of HRV, as measured by SDNN.

**FIGURE 4:**
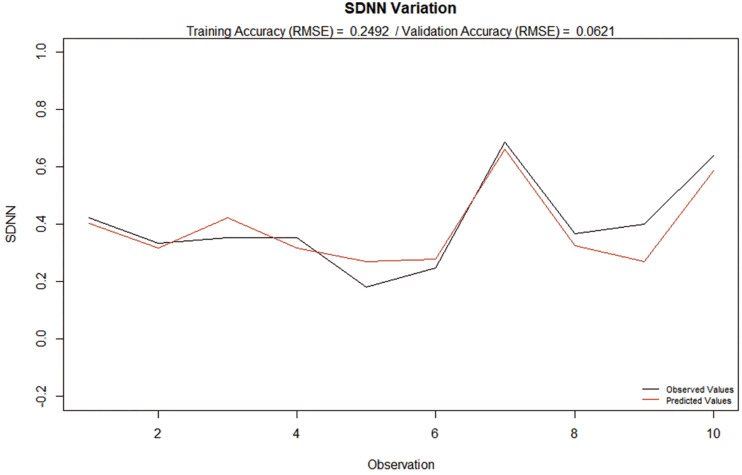
Observed and predicted SDNN Ratio based on the variation of CD16(%), CD66b (MFI), MPO(%), MPO (MFI), Annexin +, CD66b +, CD31 + and CD41 +. Application of the second model on an out-of-sample data set.

In order to test the robustness of the modeling process, further tests were performed. Firstly, a second model was created, where the calibration of the RBF-NN was done using only a portion of the data (*N* = 37) and, then, it was applied to the remaining 10 observations in an out-of-sample validation process, as previously described (result illustrated in Figure 4). Interestingly, the training accuracy was slightly higher than the first model, even with the use of a smaller dataset for training in comparison with the in-sample applied model display in Figure 2. The similarity of the Training and Validation Accuracies reinforces the predictive capability of the model, while providing support that the model was not overfitted.

**FIGURE 5:**
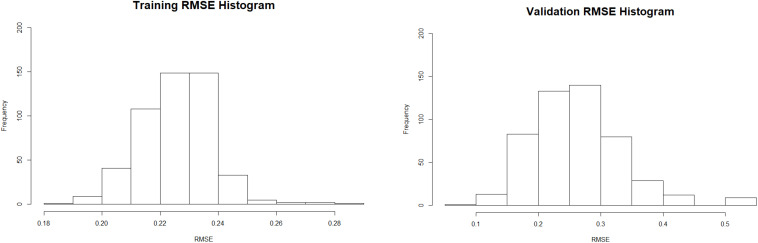
Training and validation error distribution produced by the 500 models created based on the resampled data.

Secondly, through the utilization of a stochastic resampling technique, 1.000 new datasets were created divided in: (i) 500 containing part of the data (*N* = 37) (training datasets) and (ii) 500 containing the remaining part of the data (*N* = 10), that were not used in the respective training dataset creation (validation datasets). Again, Gaussian functions were used for activation and each model was based on the neural network that produced the best accuracy for each training set in 100 trials, in a stochastic process of centroids determination, and subsequently applied to the respective validation dataset. This process was used to estimate the distribution of the error of the modeling process when applied to different training and validation datasets (Figure 5).

The data above can be summarized by the mean error in the training and validation process. Similarly to the model demonstrated above, the mean error observed during the training of the RBF-NN is lower than the mean error observed in the application of the model to the validation datasets (respectively 0.22 and 0.26), reinforcing prediction capabilities of the model and the relationship between the observed variables.

All the data was normalized, according to the normalization method described in the Materials and Method section.

## Discussion

Although the role of the inflammatory process associated with decompression has gained support in the past decade (Møllerløkken and Brubakk, 2009; Madden and Laden, 2009, the perception that decompression sickness is mainly due to the appearance and growth of bubbles is still very much accepted. A series of three articles recently published correlate symptoms of decompression sickness to tissue perfusion alterations that, ultimately, causes the pressure of inert gas to raise enough to produce bubbles in a specific location (Lu et al., 2018; Strauss et al., 2018a, b). Concomitantly, the possibility of microparticles acting as nucleation points, which would link the observation of microparticles to bubbles, is still under investigation and cannot be ruled out (Thom et al., 2013a).

The contributions of MPs to the inflammatory process are becoming progressively better understood. The release of platelet, endothelial and leukocyte MPs is increased during inflammatory conditions (Cognasse et al., 2015). Activation by inflammatory cytokines, ROS and stimulus TNF-α is known to result in the release of endothelial-derived microparticles (CD31 +). Oxidative stress is known to cause the release of CD31 + MPs, which ultimately attract leukocytes to the inflammatory site by adhesion molecules.

Activated platelets release microparticles (CD41 +) that might work as a mechanism for intercellular (endogenous) signaling by inducing immune responses in distant sites. Our results demonstrate an increase in the number of circulating CD41 + MPs after decompression. This pattern is compatible with the reduction in the numbers of circulating platelets, hypothesized to reflect their activation and recruitment to inflammation sites (Table 2).

The results obtained in the present study demonstrated that MPs subtypes have little and not statistically significant correlation among each other, which contradicts another study previously published (Thom et al., 2015). The reason for contradiction would require further investigation. In the same line, the post dive magnitude of all MPs subtypes mean increase reported by the same study is greater than the mean increase observed in the present study.

Interestingly, leukocyte-derived microparticles (CD66b +) appears to carry an endogenous anti-inflammatory effect (Batool et al., 2013). We observed a significant increase in their levels in the post decompression dataset. It is worth to note that, although a causal relationship cannot be established, other inflammatory markers alterations and the different levels of CD66b + MPs (i.e., MPO) in the different experimental protocols offers support to infer an anti-inflammatory role of the CD66b +. Due to the lack of specific controls, this study can only speculate that the higher levels of CD66b + MPs observed are associated with the post-dive lower levels of MPO detected in some cases.

In a contrary direction, however, a study designed to evaluate the association of microparticles and decompression sickness reported a post-dive 30-fold increase in CD66b + MPs, even in the absence of decompression sickness symptoms, causing the anti-inflammatory role this microparticle subtype speculated above to be void and demonstrating a high positive correlation between MPO and leukocyte-derived MPs (Thom et al., 2015).

We observed in a control group in our previous study that the exposure to high fractions of oxygen, even in the absence of changes in the ambient pressure, is associated with increased Heart Rate Variability measured, given by SDNN in the time domain and in the HF in the frequency domain (Schirato et al., 2018). It is plausible that general post dive increased values in these HRV indicators observed in this (and in other previously published studies) is related to the hyperoxia almost inherently linked to the exposure to hyperbaric environments. Interestingly, in the subjects exposed to increased hydrostatic pressure, a post decompression increase in the Low Frequency was observed, while the total power observed was not necessarily greater, due to a reduction in the High Frequency. However, this change in the LF/HF ratio does not necessarily translates in changes in the sympatho-vagal balance (Billman, 2013).

In regard to HRV and decompression sickness, two studies in swine model from the same research group reported contradictory results. One study reported a reduction in the two branches of the Autonomic Nervous System (ANS) activity, when symptoms of decompression sickness are present (Bai et al., 2009), while the second reported an elevated parasympathetic activity, with increased power associated with the High Frequency (Heathers, 2014), during the development of cardio vascular decompression sickness (characterized by breathing difficulties, hemoptysis, dyspnea and considered a serious manifestation of decompression sickness), as well as a decrease in the sympathetic branch activity when compared to the baseline pre dive values (Bai et al., 2013). Moreover, it does demonstrate that as the time after the end of decompression increases, a decrease in power in the frequency domain bands of HRV happens in the group diagnosed with decompression sickness in comparison with the control group, affecting especially the sympathetic tone, with impact in the overall variability measured by SDNN. It is worth to note that the experimental protocols adopted by the two studies, in terms of exposure, were not comparable and that the animals used in the first study, that reported reduction in the activity of the ANS, received diazepam. Diazepam is known to be associated with an overall reduction of Heart Rate Variability in humans and it appears to be a fair assumption that it could cause a similar reduction in swine (Agelink et al., 2002), potentially affecting the results reported in the study.

In the present study, a correlation between HRV observed after decompression, with other indicators of physiological stress known to be associated with decompression was made. In this context, physiological stress might be defined as the inflammation and activation of the immune system processes that are, apparently, tied to the oxidative stress caused by the compression-decompression process (Thom et al., 2012, 2017).

The results here obtained demonstrated that HRV indicators, in special SDNN, as a broader indicator of variability in the time domain, and the HF in the frequency domain, are significantly negatively correlated with different markers of inflammation and immune system activation provoked by the exposure to hyperbaric environments and subsequent decompression. Increased expression of myeloperoxidase, as well as increased number of circulating Annexin + MPs are associated with reduced variability, either measured by SDNN, or by power associated with the sum of all frequencies, approximated by *SDNN*^2^. High Frequency is known to have a higher correlation with the total variance and, as expected, demonstrated inverse correlations with the number of circulating MPs as well. On the other hand, the post dive LF values were consistently and significantly higher than the pre dive values. In this study, a significant association between LF and different markers of inflammation and immune system activation was not observed, although previous studies have reported a negative association between Low Frequency and inflammatory parameter (Ernst, 2017). A possible explanation for this fact might be that endothelial dysfunction (an usual observation after decompression) causes changes in the baroreceptor activity. Low Frequency is intimately linked to baroreflex function, as previous studies demonstrated that carotid sinus stimulation increases LF power in individuals with normal baroreflex function, but not in those with impaired baroreflex sensitivity (Rahman et al., 2011; Chapleau et al., 2016).

Frequency domain components of HRV were reported to be inversely correlated with inflammation markers by at least one large scale study (Sloan, 2015). Additionally, a review of thirteen studies about HRV and inflammation, in the same line, reported an inverse relationship between inflammatory markers and the time and frequency domain indicators of HRV in cardiovascular disease (Haensel et al., 2012).

In the vasculature, MPO-generated HOCl has been shown to affect eNOS activity and to decrease NO bioavailability, thereby negatively affecting endothelial function. Moreover, MPO was demonstrated to be associated with neutrophils activation, through the activation of intracellular signaling cascades, which increase ROS production. A previous study that evaluated MPO levels in divers suffering from decompression sickness and in a control group, reported a significant increase in both percentage of MPO expression (MPO + %) and mean fluorescence intensity (MPO MFI) in divers diagnosed with decompression sickness (Thom et al., 2015).

Based on the above, reductions in HRV, in special overall variance, given by SDNN and Higher Frequency, might be associated with increased levels of decompression-related physiological stress. The accuracy of the model created to predict the pre and post dive rate of variation of the SDNN based on variation of inflammatory markers and number of circulating MPs (Figures 2, 3) provides further evidence of the association between physiological stress markers and HRV, corroborating the understanding that it might be a good estimator of decompression-related physiological stress.

As for the LF, the increased values observed in this and in previous studies appear to be less likely to be related with increased pressures of inspired oxygen, and more likely to associated with the endothelial dysfunction usually associated with decompression-induced physiological stress. The positive association observed in the results presented in this study between LF and CD31 + MPs (*p*-value = 0.08), provides some support to this understanding. A more extensive discussion of the relationship between Heart Rate Variability and endothelial function in SCUBA diving is presented elsewhere (Ernst, 2017).

Heart Rate Variability presents some advantages when compared to blood and biochemical analysis: data can be easily obtained through a non-invasive procedure, at a relatively low cost, comparatively simple infrastructure, and results can be obtained almost instantaneously. For those reasons, finding an association between HRV and inflammatory markers and physiological stress under post-decompression context provides an opportunity to efficiently access the inflammatory state of the diver as she emerges.

### A Note on the Subject Who Developed Decompression Sickness

Despite the above-reported relationship of SDNN and HF with some markers of physiological stress, such a negative correlation might not hold true in cases where the clinical conditions of decompression sickness arise. In the one single case of decompression sickness diagnosed during this study, an increase in the SDNN and in the High Frequency of the spectrogram was observed, notwithstanding the significant increase in all markers of inflammation and immune system activation, in special the MPO values (post experiment MPO expression was 1.4x the pre experiment value).

One possible explanation for this different HRV pattern might be the symptoms associated with decompression sickness reported by the volunteer, still during the ECG recording section. It could be expected that, whenever there are manifestations of symptoms related to decompression sickness, not only the pain, but the anxiety experimented by the individual at this point, would directly affect diverse physiological variables, among them ventilatory rate and blood pressure, thus indirectly affecting HRV as well. Not to mention potential decompression sickness related neurological impairment that might cause other alterations on its own. Interestingly, the results observed in this case, where decompression sickness symptoms were present, are comparable with the ones published by one study that submitted animals to provocative decompression (Bai et al., 2013) and reported increased SDNN, Higher Frequency and parasympathetic activity in animals suffering from cardiovascular decompression sickness. The same study reported a progressive loss of HRV in the animal group as the time after decompression passed. In the present study, the EGC recordings were taken in one single interval (as described in the Material and Methods section), therefore it is not possible to speculate how HRV indicators would behave more than one hour after the end of the decompression.

### Limitations of the Present Study

Heart Rate Variability is highly unsteady and there is an important variance in its estimators, such as the standard deviation of the interval between consecutive heartbeats (SDNN), both among and within subjects. As expected, a large inter-individual variance in the responses to decompression was observed in the present study. This, in addition to the fact that the study was produced with a relatively small cohort, leads to caution when the statistical significance of the results is analyzed. Besides, it is important to note that most of the data was obtained through dives simulated in hyperbaric chambers, which might not necessarily reflect the actual conditions encountered when divers are immersed (Vann, 2004). Additionally, different breathing apparatus were used in different facilities, therefore with different work of breathing (the resistance to breathe caused by the equipment), which may have impacted the observed results.

## Data Availability Statement

The raw data supporting the conclusions of this article will be made available by the authors, without undue reservation.

## Ethics Statement

The studies involving human participants were reviewed and approved by Ethical committee of the Biosciences Institute of the University of São Paulo approved the experimental protocol CAAE #91231618.6.0000.5464. The patients/participants provided their written informed consent to participate in this study.

## Author Contributions

All authors listed have made a substantial, direct and intellectual contribution to the work, and approved it for publication.

## Conflict of Interest

The authors declare that the research was conducted in the absence of any commercial or financial relationships that could be construed as a potential conflict of interest.

## Figures and Tables

**Appendix 1:**
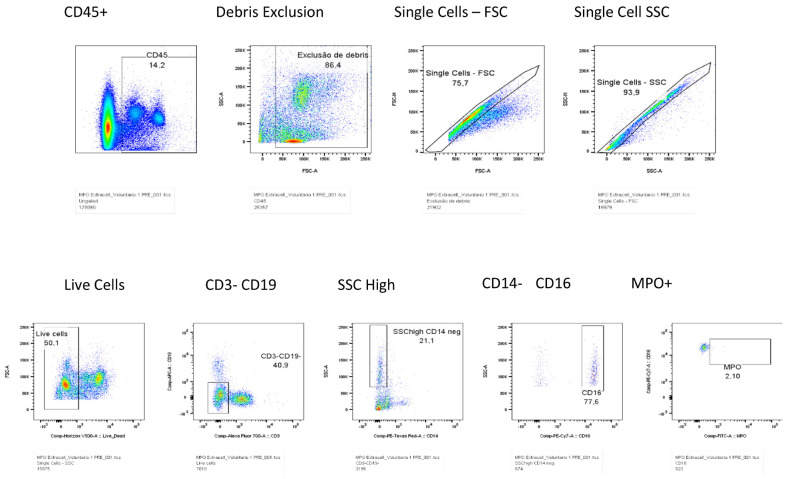
Flow cytometry gates definition Immunophenotyping.

**Appendix 2:**
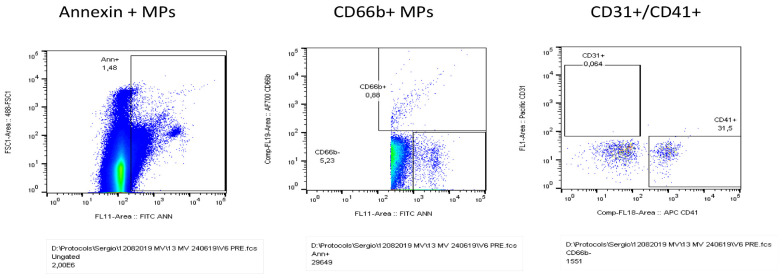
Flow cytometry gates definition for Microparticles analysis.
